# Symptomatic Colitis in a Patient with Dengue Fever: A Case Report

**DOI:** 10.4269/ajtmh.19-0217

**Published:** 2020-09

**Authors:** Avisnata Das, Indrajeet Kumar Tiwary, Mahesh Kumar Goenka

**Affiliations:** Institute of Gastrosciences and Liver, Apollo Gleneagles Hospital, Kolkata, India

Dengue, a challenging arboviral disease^1^ prevalent in the tropics, is very rarely known to present with acute colitis, with our literature search showing only two such cases^2,3^ reported worldwide so far. Although upper gastrointestinal involvement in dengue fever is reported more frequently, including upper GI bleeding with endoscopic evidence of the same,^4,5^ colonic involvement and colonoscopy-proven colitis are very rare. Here, we describe the case of a patient with dengue fever who presented with acute colitis and hematochezia and was treated by our department.

A 34-year-old normotensive, nondiabetic male patient was admitted with a history of fever, pain in the abdomen, and passage of fresh blood with stools for 4 days. At admission, the patient was afebrile, normotensive, and mildly tachycardic. Initial investigations were significant for thrombocytopenia (platelet count = 60,000/cu mm), hyponatremia (Na = 130 mmol/L), mild jaundice (total bilirubin = 1.8 mg/dL), and deranged liver enzymes. Dengue NS1 was negative but dengue IgM and dengue IgG were both positive. Malaria dual antigen (*Plasmodium vivax* LDH and *Plasmodium falciparum* HRP-2) and peripheral smear, Typhi Dot IgM, *Cytomegalovirus* IgM, and viral hepatitis markers (HBsAg, anti-HCV antibody, IgA anti-HAV, and IgM anti-anti Hepatitis E antibody) were all negative. No history of hemorrhagic rash or bleeding (except bleeding P/R) was present.

**Figure 1.**
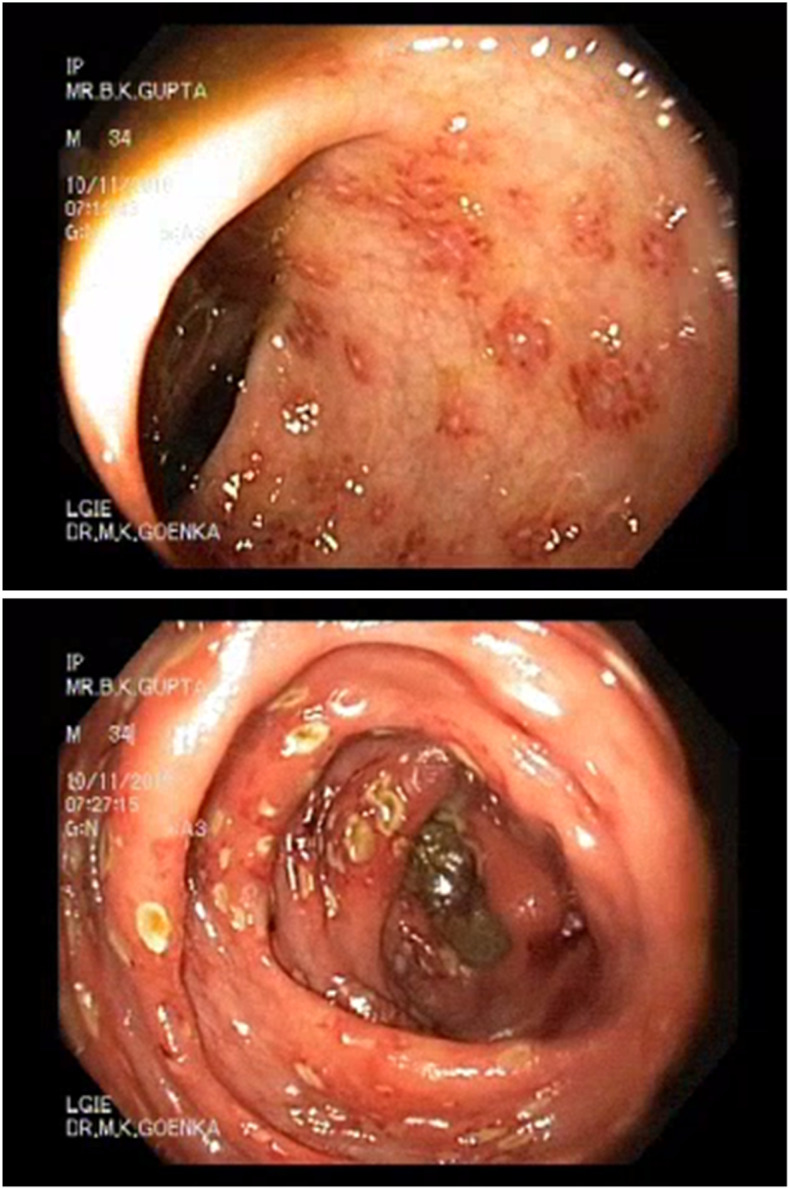
Colonoscopic images showing evidence of colitis. Colonoscopy was performed during admission of the patient. This figure appears in color at www.ajtmh.org.

The patient underwent colonoscopy which showed multiple aphthoid ulcers throughout the colon, more predominant in the rectosigmoid area and cecum, with the presence of perilesional edema (Figure 1). The findings pointed at possible infective colitis. Histopathology from cecal ulcer biopsy specimen showed ulcerated colonic mucosa with focal cryptitis and moderate mixed inflammation of the lamina propria (suggestive of active colitis). Upper gastrointestinal endoscopy was normal.

**Figure 2.**
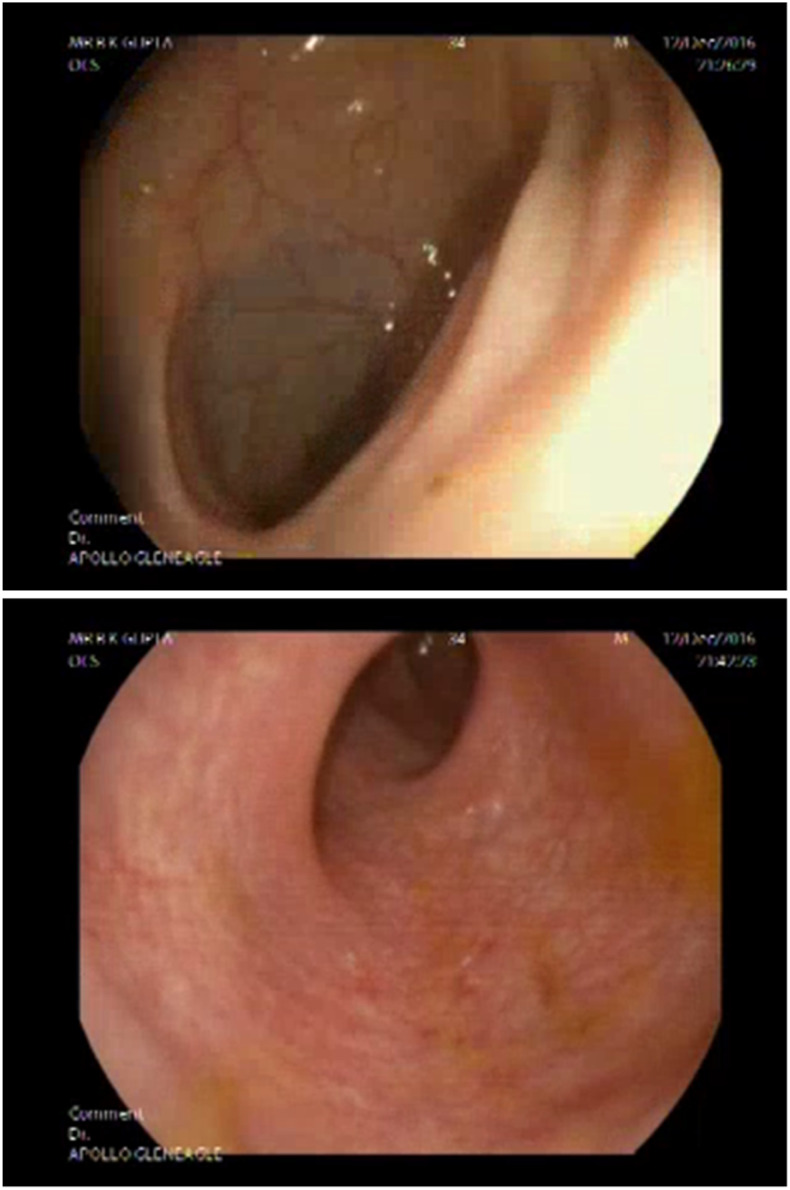
Near-normal colonoscopic appearance of the patient 6 weeks after discharge. The resolution of colitis coincided with the clinical remission of the patient. This figure appears in color at www.ajtmh.org.

The patient responded well to conservative management with IV fluids, antipyretics, and supportive measures, and was discharged when appropriate (no Dengue hemorrhagic fever or Dengue shock syndrome was noted). Repeat colonoscopy at 6 weeks showed resolving colonic ulcers and normalizing mucosa (Figure 2).

The colonoscopic impression and histopathologic findings strongly pointed toward infective colitis. Nevertheless, possible differentials including inflammatory bowel disease, vasculitis, and bacterial infection were considered. Fecal calprotectin was negative (not in favor of IBD); there was no rash, mucosal involvement, typical pattern of arthropathy, and negative antinuclear antibody (not in favor of vasculitis), and no antibacterial therapy was required for remission (not in favor of bacterial colitis). Moreover, the temporal relation between the onset of fever and colitis with concurrent dengue IgM positivity and the resolution of colitis following the resolution of dengue fever also fortified dengue colitis as the primary clinical diagnosis. No specific treatment was given for the colitis between discharge and the follow-up colonoscopy at 4 weeks, as it was a colitis of suspected viral etiology.

Our case illustrates an interesting and rare presentation of dengue fever where the patient had satisfactory clinical outcomes and colitis showed resolution with dengue remission.
